# Group 3 innate lymphoid cells secret neutrophil chemoattractants and are insensitive to glucocorticoid via aberrant GR phosphorylation

**DOI:** 10.1186/s12931-023-02395-5

**Published:** 2023-03-23

**Authors:** Li Xiu He, Ling Yang, Ting Liu, Yi Na Li, Ting Xuan Huang, Lan Lan Zhang, Jian Luo, Chun Tao Liu

**Affiliations:** 1 Department of Respiratory and Critical Care Medicine, Clinical Research Center for Respiratory Disease, West China Hospital, Sichuan University, Chengdu, 610041 China; 2 Department of Respiratory and Critical Care Medicine, The Affiliated Hospital of Guizhou Medical University, Guiyang, 550001 Guizhou China; 3 Clinical Trial Center, National Medical Products Administration Key Laboratory for Clinical Research and Evaluation of Innovative Drugs, West China Hospital, Sichuan University, Chengdu, 610041 China; 4 Department of Pulmonary and Critical Care Medicine, The First Affiliated Hospital of USTC, Division of Life Sciences and Medicine, University of Science and Technology of China, Hefei, 230001 Anhui China; 5 Respiratory Medicine Unit and National Institute for Health and Care Research (NIHR) Oxford Biomedical Research Centre, Nuffield Department of Medicine, Experimental Medicine, University of Oxford, Oxford, OX3 9DU UK

**Keywords:** Asthma, Neutrophil chemoattractant, Glucocorticoid resistant, Group 3 innate lymphoid cells, Neutrophilic inflammation

## Abstract

**Background:**

Patients with neutrophil-mediated asthma have poor response to glucocorticoids. The roles and mechanisms of group 3 innate lymphoid cells (ILC3s) in inducing neutrophilic airway inflammation and glucocorticoid resistance in asthma have not been fully clarified.

**Methods:**

ILC3s in peripheral blood were measured by flow cytometry in patients with eosinophilic asthma (EA) and non-eosinophilic asthma (NEA). ILC3s were sorted and cultured in vitro for RNA sequencing. Cytokines production and signaling pathways in ILC3s after IL-1β stimulation and dexamethasone treatment were determined by real-time PCR, flow cytometry, ELISA and western blot.

**Results:**

The percentage and numbers of ILC3s in peripheral blood was higher in patients with NEA compared with EA, and negatively correlated with blood eosinophils. IL-1β stimulation significantly enhanced CXCL8 and CXCL1 production in ILC3s via activation of p65 NF-κB and p38/JNK MAPK signaling pathways. The expression of neutrophil chemoattractants from ILC3s was insensitive to dexamethasone treatment. Dexamethasone significantly increased phosphorylation of glucocorticoid receptor (GR) at Ser226 but only with a weak induction at Ser211 residues in ILC3s. Compared to human bronchial epithelial cell line (16HBE cells), the ratio of p-GR S226 to p-GR S211 (p-GR S226/S211) was significantly higher in ILC3s at baseline and after dexamethasone treatment. In addition, IL-1β could induce Ser226 phosphorylation and had a crosstalk effect to dexamethasone via NF-κB pathway.

**Conclusions:**

ILC3s were elevated in patients with NEA, and associated with neutrophil inflammation by release of neutrophil chemoattractants and were glucocorticoid (GC) resistant. This paper provides a novel cellular and molecular mechanisms of neutrophil inflammation and GC-resistance in asthma.

*Trial registration* The study has been prospectively registered in the World Health Organization International Clinical Trials Registry Platform (ChiCTR1900027125)

**Supplementary Information:**

The online version contains supplementary material available at 10.1186/s12931-023-02395-5.

## Background

Group 3 innate lymphoid cells (ILC3s) have crucial roles in immunity and tissue homeostasis [1, 2], which are widely distributed throughout the body and are constitutively present at mucosal barrier sites such as the lung, liver, gut, spleen, skin and secondary lymphoid tissues [3]. ILC3s lack the diversified antigen receptors expressed on T cells and B cells and are defined by expression of the Retinoid-related orphan receptorγt (RORγt) and the secretion of IL-17, IL-22 in response to IL-23 and IL1-β [4, 5]. A recent study found ILC3 (IL-17^+^RORγt^+^) cells were responsible for the development of airway hyperreactivity (AHR) induced by IL-1β in obese mice asthma model, and blockade of IL-1β abolished the AHR and reduced the number of ILC3 cells [6]. Meanwhile, these ILC3s were found to be increased in non-allergic neutrophilic asthma mice [7] and a similar IL-17^+^ ILCs were also found in bronchoalveolar lavage fluid from human patients with severe asthma[6]. However, the roles of ILC3 in asthmatic patients with airway neutrophilic inflammation are still not clear.

Asthma is a heterogeneous disease with two broad inflammatory phenotypes: eosinophilic asthma (EA) and non-eosinophilic asthma (mainly mediated by neutrophil) (NEA) [8, 9]. Neutrophil mediated asthma occurs in about 50% of corticosteroid resistant/insensitive asthma cases [10]. Evidence suggests IL-17 produced by Th17 cells appears to drive neutrophil-predominant steroid-resistant asthma [11, 12] and increases the production and release of chemokine IL-8 via airway epithelial cells, further propagating the chemotactic neutrophil response [13]. Since ILC3s can mimic the function of T helper type 17 (Th17) cells [1], they may also contribute to corticosteroid resistance in asthma, which is supported by a recent study that found the proportions of lung ILC3s in a murine neutrophilic asthma model was not altered by fluticasone propionate treatment [7]. However, the mechanisms behind the ILC3 associated steroid resistance are unknown. Therefore, in this study we investigated the relationship between ILC3s and neutrophilic inflammation in asthma patients and the mechanisms of corticosteroid resistance, which will provide a better understanding of the innate immunity and treatment target for NEA.

## Methods

### Human subjects

Adult patients with asthma were consecutively recruited from Respiratory Outpatient Department of West China Hospital, Sichuan University from June 2019 to December 2020, according to diagnosis of Global Initiative for Asthma (GINA) recommendations [14]. Patients were stratified by baseline blood eosinophil counts 300/μL or greater and less than 300 cells/μL as eosinophilic asthma (EA) and non-eosinophilic asthma (NEA), respectively [15, 16]. Detailed inclusion and exclusion criteria for subjects were described as previously reported [17]. The healthy control population enrolled healthy volunteers from West China Hospital. The study was approved by the Institutional Review Board (IRB) at West China Hospital, Sichuan University (Chengdu, China) (No. 2019–856). All participants provided written informed consent.

### Flow cytometry analysis of peripheral blood

Fresh blood was collected in EDTA-treated tubes and peripheral blood mononuclear cells (PBMCs) were isolated as previously reported [18, 19] and stained with Zombie Aqua (Live/Dead) together with antibody panel 1(Additional file 4: Table S1). Lineage (Lin) markers included CD19/CD56/CD14/CD11b/CD11c/CD123/FcεRI. Group 2 innate lymphoid cell (ILC2) was defined as Lin^−^CD45^+^CD3^−^CD4^−^CD8^−^CD127^+^CRTH2^+^, Group 1 innate lymphoid cell (ILC1) was defined as Lin^−^CD45^+^CD3^−^CD4^−^CD8^−^CD127^+^CRTH2^−^CD117^−^ and ILC3 was defined as Lin^-^CD45^+^CD3^−^CD4^−^CD8^−^CD127^+^CRTH2^−^CD117^+^ [20–22] (Additional file 1: Fig. S1).

### Cell lines and reagents

Human bronchial epithelial cell line (16HBE cells) were received as a gift from Tianfu Life Science Center of Sichuan University West China Hospital. Human IL-2, recombinant human IL-1β and IL-23 were purchased from Peprotech. NF-κB inhibitor (TPCA-1), p38 inhibitor (SB203580) and JNK inhibitor (SP600125) were purchased from Selleck. Dexamethasone was purchased from Sigma.

### ILC3s sorting and culture

PBMCs from healthy donors were labeled with the antibody panel 2 (Additional file 4: Table S1) after CD3 depletion by using CD3 Microbeads (Miltenyi Biotech). ILC3 cells were sorted with the FACSAria III (BD, Franklin Lakes, NJ).

The cells were cultured in RPMI with 10% human serum, 1× l-glutamine, 1× penicillin/streptomycin, 1× sodium pyruvate, 1× nonessential amino acids, and 250 U/mL IL-2. Details were described in our previously published study [23].

### Intracellular staining

For RORγt staining, cultured ILC3s were incubated with antibody for RORγt after surface staining with Zombie Aqua (Live/Dead) together with antibody panel 3 (Additional file 4: Table S1) and fixation/permeabilization with FOXP3/Transcription Factor Staining Buffer set (eBioscience) [24]. ILC3 was defined as CD3^-^RORγt^+^. Cells with purity ≥ 90% were used for subsequent experiments.

For staining of IL-22, IL-17A and CXCL8, cultured ILC3s were stimulated with Phorbol 12-myristate 13-acetate (PMA) cocktail in the presence of Brefeldin A (Cell Activation Cocktail with Brefeldin, Biolegend) for 6 h at 37 ℃. After staining with Zombie Aqua (Live/Dead) and fixation with IC Fixation Buffer (eBioscience), cells were permeabilized with a permeabilization buffer (Invitrogen) and labeled with corresponding antibodies (Additional file 4: Table S1). The above samples were acquired on a BD LSR II flow cytometer.

### RNA extraction and quantitative real-time polymerase chain reaction (PCR)

Total RNA was extracted from ILC3 cells using RNeasy Mini Kit (QIAGEN) following the manufacturer’s instructions and reverse-transcribed to cDNA using HiScript III RT SuperMix for qPCR (+gDNA wiper) Kit (Vazyme, China). Primers sequences were presented in Additional file 4: Table S2. Data correction was performed using Bio-Rad CFX Maestro and analyzed by 2^−ΔΔCt^ method.

### RNA sequencing (RNA-seq)

ILC3s were stimulated with or without IL-1β + IL-23 for 48 h before sending to Beijing Novogene Bioinformation Technology Co., Ltd. (Beijing, China) in TRIzol Reagent for RNA-Seq. Standard methods were used to extract RNA, and RNA integrity was assessed using the RNA Nano 6000 Assay Kit of the Bioanalyzer. 1 μg RNA was used for cDNA library preparation after purification using Poly-T oligo-attached magnetic beads. Sequencing was performed on the Illumina Novaseq 6000 (Novogene Bioinformatics Technology Co., Ltd., Tianjin, China) platform [25].

### Enzyme-linked immunosorbent assay (ELISA)

Cell supernatants were analyzed by Human CXCL8 Uncoated ELISA kit (Thermo Fisher Scientific) and human CXCL1 ELISA kit (Elabscience, China) following manufacturer’s recommendations.

### Western blot

Proteins of cells were extracted with radioimmunoprecipitation (RIPA) lysis buffer (Beyotime, China) with phenylmethanesulfonyl fluoride (PMSF) and protease inhibitor cocktail and quantified by BCA Protein Assay Kit (Beyotime, China). The process was performed as previously reported [26]. Antibody and antigen complexes were detected using ECL chemiluminescent kit (Beyotime, China) by ChemiDoc™ MP Imaging System (Bio-Rad, USA). Antibodies used in western blot were presented in Additional file 4: Table S3.

### Statistical analysis

Continuous variables are expressed as mean ± standard deviation (SD) or median (interquartile range, IQR). Categorical variables are summarized as frequencies and proportions. Differences between groups were analyzed with Student’s t test or Mann–Whitney test. And for three or more groups, Analysis of Variance or the Kruskal–Wallis test were performed, Tukey HSD (Tukey Honest Significant Differences) were used for multiple comparisons as post hoc test. Categorical data were compared using Chi-square test or Fisher exact test. Test of Spearman was performed for evaluating correlations. Differences were considered statistically significant at ^*^P < 0.05, ^**^P < 0.01, ^***^P < 0.001 and ^****^P < 0.0001. Data was analyzed by Prism 6.0 (GraphPad) and flow cytometry data was analyzed by FlowJo 9.7.6 software (Tree Star).

For sequencing data, differential gene expression analysis was performed using the DESeq2 R package (1.20.0). The *P*-value was adjusted using the Benjamini & Hochberg method for controlling the false discovery rate. Genes with adjusted *P*-value < 0.05 and absolute value of log2(FoldChange) ≥ 1 were assigned as differentially expressed genes. Gene Ontology (GO) enrichment analysis and KEGG pathways analysis of differentially expressed genes were conducted by the clusterProfiler R package, and terms with adjusted *P-*value < 0.05 were considered significantly enriched.

## Results

### ILC3 counts are higher in patients with NEA and they show a negative correlation with blood eosinophils

**Fig. 1:**
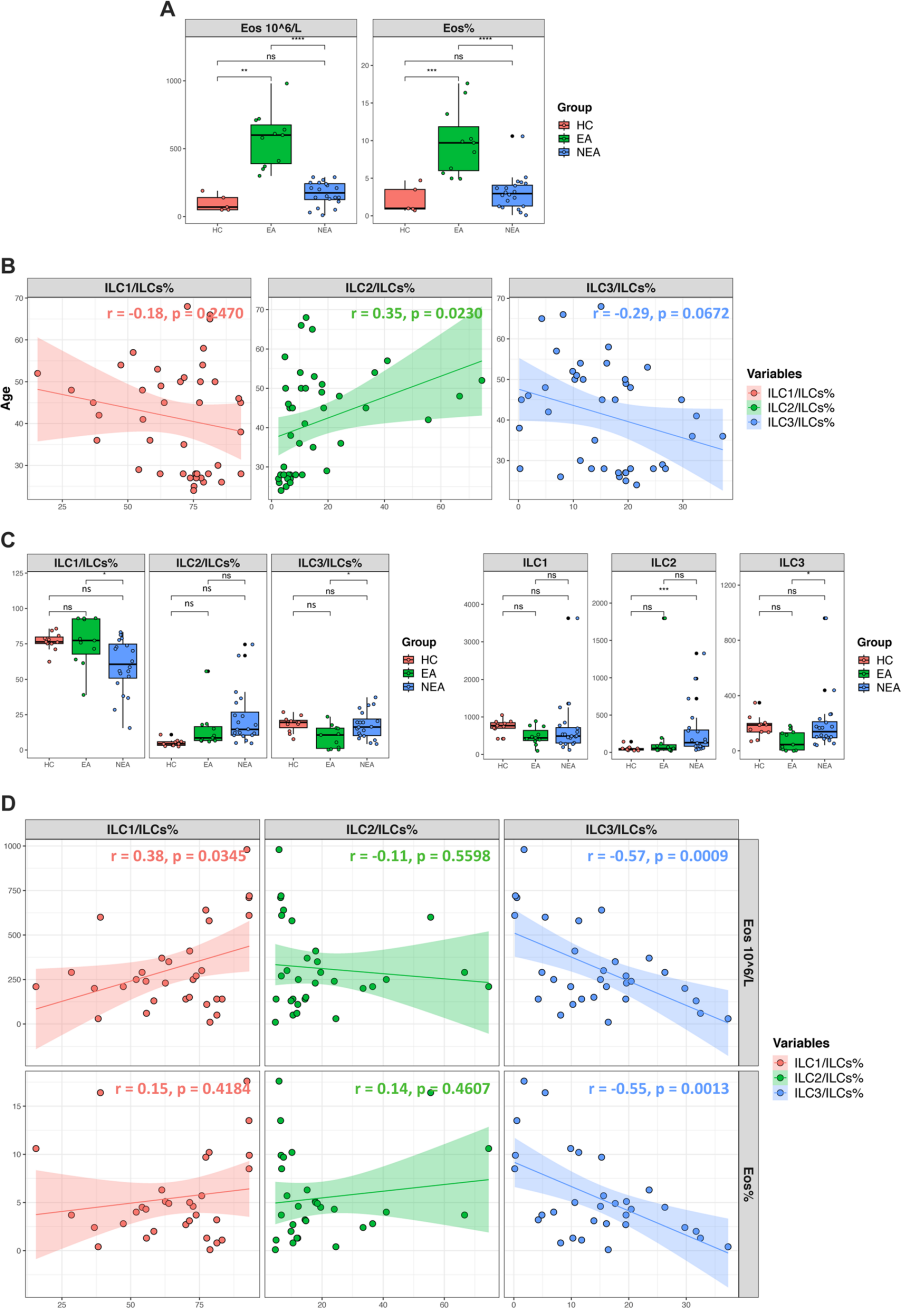
ILC3s elevated in patients with NEA compared to EA. **A** Blood eosinophils count and percentage among healthy control (HC) and patients with NEA and EA. **B** Correlation between subsets of ILCs and age. **C** Percentage and numbers (in every 50 × 10^5^ recorded lymphocyte) of ILC1, ILC2 and ILC3 in HC, NEA and EA patients. **D** Correlation between ILC subsets and blood eosinophils count and percentage. ****p < 0.0001, ***p < 0.001, **p < 0.01, *p < 0.05, ns p > 0.05

Twenty patients with NEA and eleven patients with EA were enrolled in this study, respectively. And eleven healthy donors were included as healthy control (HC). In the NEA group, blood eosinophils count (170.00 ± 87.12 × 10^6^/L vs 570.00 ± 201.15 × 10^6^/L, *P* < 0.001) and percentage (2.95 [1.30, 4.23]% vs 9.70 [5.70, 13.50]%, *P* < 0.001), fractional exhaled nitric oxide (FeNO) (54.00 [27.25, 68.25] ppb vs 87.00 [60.00, 153.0]ppb, *P* = 0.032) were lower compared to the EA group (Table 1, Fig. 1A), and the percentage of blood neutrophils was higher (Additional file 2: Fig. S2A). To determine the difference of ILC subsets between EA and NEA, the proportions of ILC subsets in peripheral blood were measured (Additional file 1: Fig. S1). Due to the imbalance of age between HC and patients with asthma, we firstly checked the relationship between age and ILC subsets and found a negative correlation with ILC3 (r = − 0.29, *P* = 0.067) and positive correlation with ILC2 (r = 0.35, *P* = 0.023) but not ILC1(Fig. 1B). After adjusting for age, the percentage (18.33 ± 5.64% vs 9.30 ± 8.16%, *P* = 0.0212) and numbers (139.00 [89.50, 224.50] vs 45.00 [2.00, 135.00], *P* = 0.021) of ILC3s in NEA group was higher than EA group (Fig. 1C). The percentage of ILC3s was negatively correlated with blood eosinophils counts (r = − 0.57, *P* < 0.001) and percentage (r = − 0.55, *P* = 0.0013) (Fig. 1D), but was not correlated with blood neutrophils counts and percentage (Additional file 2: Fig. S2B).

**Table 1:** Demographic and clinical characteristics of study subjects

Variable	NEA	EA	HC	*P*-value
N	20	11	11	
Male/Female	9/11	5/6	6/5	0.866
Age, years	48.20 ± 11.64	43.18 ± 8.84	27.00 ± 1.67^*,#^	< 0.001
BMI, kg/m^2^	23.13 ± 2.42	22.63 ± 1.62	21.85 ± 2.71	0.141
Asthma duration, years	4.00 (1.00, 10.00)	10.00 (1.50, 16.00)	–	0.640
Spirometry (Prebronchodilator)				
FEV_1_,L	1.92 (1.48, 2.85)	1.40 (0.94, 2.33)	–	0.157
FEV_1_, % predicted	79.440 ± 22.10	59.04 ± 29.21	–	0.036
FEV1/FVC	66.98 ± 12.02	58.69 ± 15.73	–	0.125
ACT score	15.40 ± 3.84	14.82 ± 2.89	–	0.665
ACQ score	2.05 ± 1.02	2.34 ± 0.50	–	0.392
Allergy, n (%)	7(35.0)	7(63.6)	–	0.132
FeNO, ppb	54.00 (27.25, 68.25)	87.00 (60.00, 153.0)	–	0.032
Blood				
Leukocytes, × 10^6^/L	6667.78 ± 2080.04	6202.22 ± 1190.51	5524.00 ± 1676.33	0.451
Eosinophils, × 10^6^/L	170.00 ± 87.12^###^	570.00 ± 201.15^***^	100.00 ± 62.45^###^	< 0.001
Eosinophils, %	2.95 (1.30, 4.23)^###^	9.70 (5.70, 13.50)^***^	1.00 (0.80, 0.10)^###^	< 0.001
Neutrophils, × 10^6^/L	3895.00 (3182.50, 4612.50)	3020.00 (2525.00, 3925.00)	3520.00 (2105.00, 4710.00)	0.254
Neutrophils,%	61.90 (57.83, 65.13)	52.7 (51.05, 58.30)	62.7 (44.90, 70.60)	0.115

Data are represented as mean ± SD, median (interquartile range, IQR) or frequency (%)

^*^P < 0.05, ^**^P < 0.005, ^***^P < 0.001 vs. NEA, with Tukey HSD test

^#^*P* < 0.05, ^##^*P* < 0.005, ^###^*P* < 0.001 vs. EA, with Tukey HSD test

ACT: Asthma Control Test; BMI: Body mass index; EA: Eosinophilic asthma; FEV_1_: Forced expiratory volume in one second; FeNO: Fractional exhaled nitric oxide; FVC: Forced vital capacity; NEA: Non-eosinophilic asthma

### IL-1β induces neutrophil chemoattractants expression in ILC3s

**Fig. 2:**
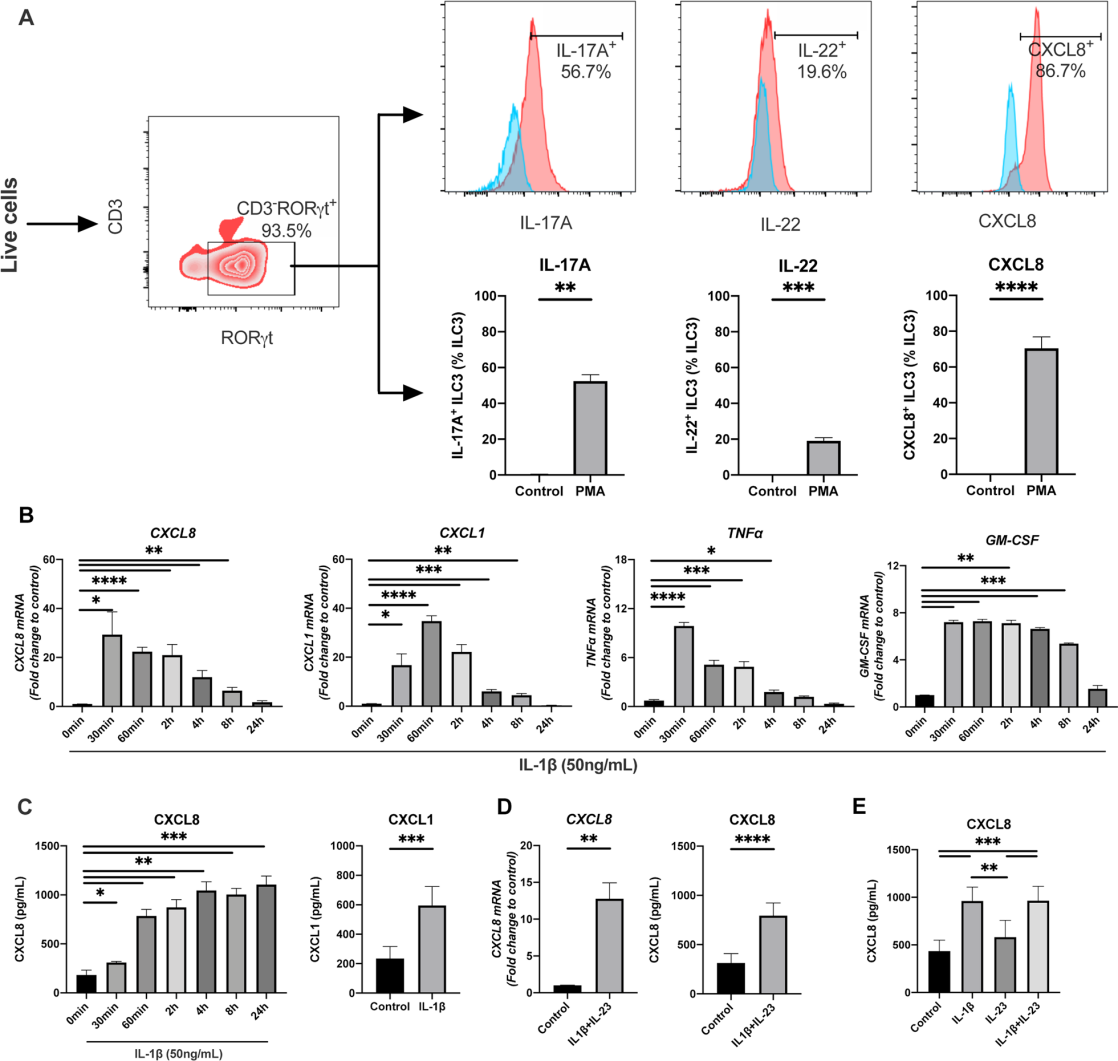
Flow cytometry gating strategy for ILC3 identification and cytokines production profiles of ILC3s. **A** ILC3s were identified by transcription factor RORγt and the secretion of IL-17, IL-22 and CXCL8. Cytokines-producing cells of ILC3s were detected by Flow cytometry. Blue, ILC3s without stimulation. Red, ILC3s stimulated with PMA + ionomycin. Numbers represent percentages of cells producing IL-17A or IL-22 or CXCL8 in ILC3s. **B** Time course of CXCL8, CXCL1, TNF-α and GM-CSF mRNA expression in ILC3s after IL-1β (50 ng/mL) stimulation. **C** Protein level of CXCL8 and CXCL1 in culture supernatant of ILC3s with or without IL-1β (50 ng/mL) stimulation after 24 h. **D**, **E** Effect of IL-1β and IL-23 on CXCL8 expression in ILC3s

In order to explore relationship between ILC3s and neutrophilic inflammation, ILC3s were sorted and cultured in vitro. Because of plasticity in vitro [27, 28], ILC3s were first identified by flow cytometry based on the transcription factor RORγt [4], and only cells with RORγt^+^ILC3 purity ≥ 90% were used for further experiments. Meanwhile, the cytokines IL-17A and IL-22 secreted by in vitro cultured cells were explored for further confirmation [5]. So, intracellular staining of transcription factor (RORγt) and cytokines (IL-17A and IL-22) by flow cytometry was performed, and results showed the expression of RORγt and production of IL-17A and IL-22 upon PMA stimulation in the in vitro cultured cells (Fig. 2A), confirming these cells were ILC3s.

Next, we measured the capability of release of neutrophil chemoattractants on ILC3s. CXCL8, one of the most potent chemokines for neutrophil migration [29, 30], was significantly enhanced after PMA or IL-1β stimulation in ILC3s at both transcriptome and protein level, peaked at 30 min and 4 h, respectively (Fig. 2A–C). In addition, IL-1β also induced production of other neutrophil chemoattractants in ILC3s including CXCL1, Tumor necrosis factor (TNF-α) and Granulocyte–macrophage colony-stimulating factor (GM-CSF) (Fig. 2B, C). Previous study reported the stimulation of ILC3s by IL-1β plus IL-23 [31–33], therefore, we sought to determine whether there is any additive or synergistic effect of IL-1β and IL-23. We found that IL-1β + IL-23 significantly increased the production of CXCL8 in ILC3s in a similar extent as IL-1β alone, and IL-23 alone did not induce CXCL8 production, indicating no additive or synergistic stimulation effect of IL-1β and IL-23 in ILC3s (Fig. 2D, E).

### ILC3s are involved in inflammation response via NF-κB and MAPK pathways

**Fig. 3:**
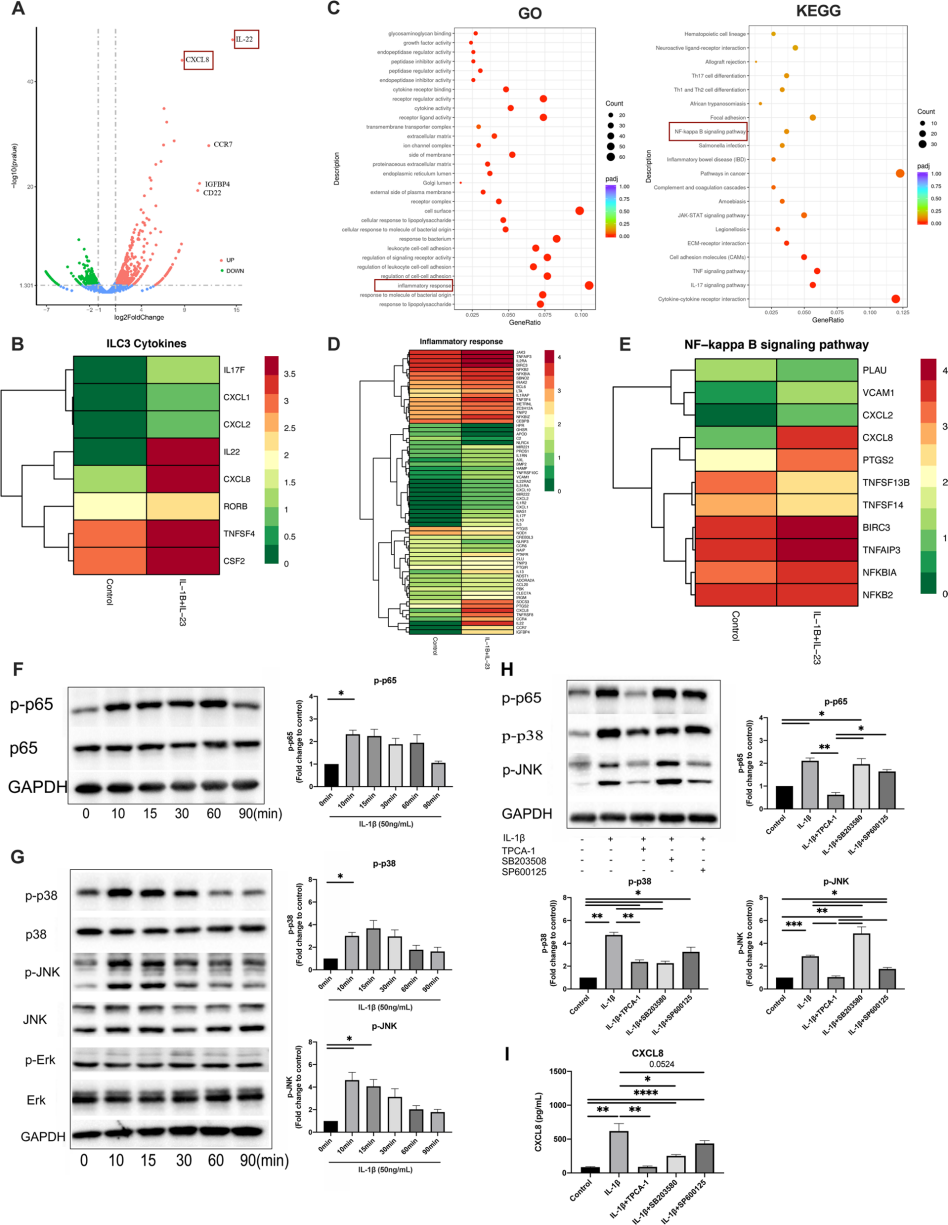
RNA sequencing of in vitro cultured ILC3s with or without IL-1β + IL-23 stimulation. **A** Volcano plots of differential expression genes (DEGs) in ILC3s stimulated by IL-1β + IL-23. The top five differential expressed genes were IL-22, CCR7, IGFBP4, CD22 and CXCL8. **B** Heatmap analysis of effector cytokines in ILC3s. In the heatmap, the redder the color is, the higher the expression is, and the greener the expression is, the lower the expression is. **C** Function terms of DEGs by Gene Ontology (GO) enrichment analysis and KEGG pathway analysis of DEGs in ILC3s upon IL-1β + IL-23(50 ng/mL) stimulation. **D** Heatmap analysis of DEGs in inflammation response. **E** The clustering heat map of DEGs involved in NF-κB signal pathway. **F**, **G** Western blot analysis by antiphospho-p65 Ab, antiphospho-p38 Ab, antiphospho-JNK Ab and antiphospho-Erk Ab. Cell extracts were prepared after stimulation with IL-1β (50 ng/mL) for indicated times. **H** Phosphorylation and inhibition of p65, p38 and JNK by specific inhibitors in ILC3s. Western blot analysis of p-p65, p-p38 and p-JNK were performed with cell extracts from ILC3s, preincubated for 1 h with p65 NF-κB inhibitor TPCA-1, p38-MAPK inhibitor SB203580 or JNK-MAPK inhibitor SP600125 and then stimulated with IL-1β (50 ng/mL) for 10 min. **I** Expression of CXCL8 protein in culture supernatant of ILC3 cells after IL-1β stimulation with or without corresponding inhibitors. ILC3s were preincubated for 1 h with indicated inhibitors and then stimulated with IL-1β (50 ng/mL) for 24 h

To investigate the pathways that regulate the production of neutrophil chemoattractants in ILC3s, RNA sequencing was performed on in vitro cultured ILC3s with or without IL-1β + IL-23 stimulation. IL-1β + IL-23 stimulation was associated with 760 up-regulated and 450 down-regulated genes, and the top five overexpressed genes were IL-22, CXCL8, CCR7, IGFBP4, and CD22 (Fig. 3A). IL-1β + IL-23 induced expression of cytokines and chemokines such as CSF2, TNFSF4, IL-22, CXCL8 and IL-17(Fig. 3B), which have been previous identified to be effector cytokines of ILC3s [5]. The GO gene set enrichment analyses showed response to inflammation was an important biological process of ILC3s (Fig. 3C, left panel), and CXCL8 was involved in “inflammatory response” during cell activation (Fig. 3D).

The KEGG pathway analysis showed the NF-κB (*P* = 0.006) (Fig. 3C, right panel) and MAPK signaling pathway (*P* = 0.071) (data not shown) were participated in ILC3s upon IL-1β + IL-23 stimulation. Further enrichment analysis also showed CXCL8 was involved in the NF-κB signaling pathway (Fig. 3E). To verify these signaling pathways, we detected the activation of key proteins in NF-κB and MAPK pathways in ILC3s by western blotting, and found IL-1β stimulation induced phosphorylation of p65, p38 and JNK, but had no effect on the activation of Erk (Fig. 3F, G). These increased phosphorylation were significantly inverted by the corresponding inhibitors (Fig. 3H). The enhancement of CXCL8 level by IL-1β was completely inhibited by p65 NF-κB inhibitor (TPCA-1) and partially inhibited by p38-MAPK inhibitor (SB203580) and JNK-MAPK inhibitor (SP600125) (Fig. 3I).

### IL-1β-induced neutrophil chemoattractants expression in ILC3s is insensitive to dexamethasone due to aberrant GR phosphorylation

**Fig. 4:**
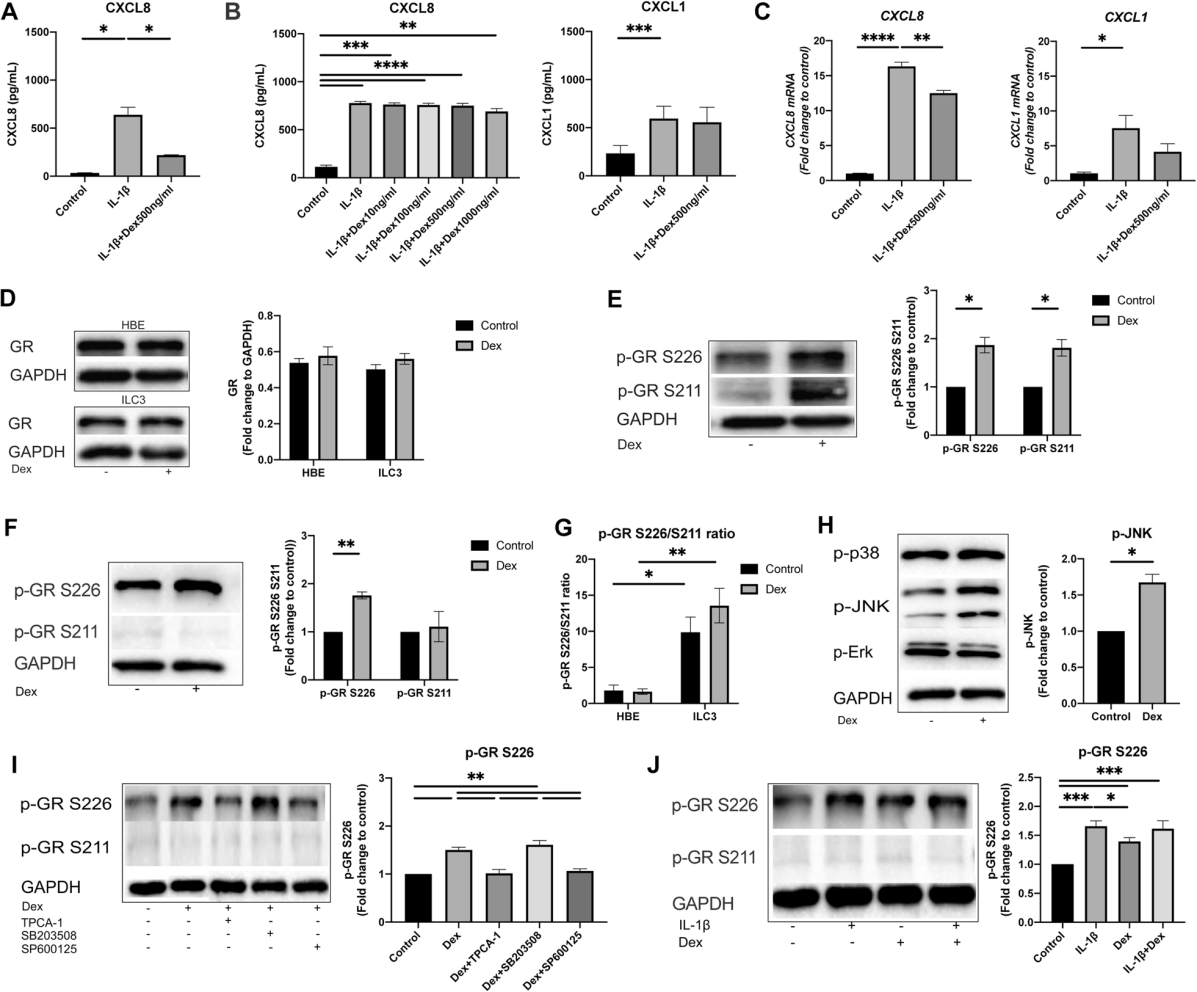
Effect of dexamethasone (Dex) on IL-1β-induced CXCL8 and CXCL1 production and phosphorylation of glucocorticoids receptor (GR) at S226 and S211 in ILC3s. **A** Expression of CXCL8 in 16HBEs with and without Dex treatment. **B**, **C**, Expression of CXCL8 and CXCL1 in ILC3s with Dex treatment by indicated concentrations. Cells were pre-treated with Dex (10 to 1000 ng/mL) for 1 h before 24 h stimulation with IL-1β (50 ng/mL), supernatants were collected and assayed for CXCL8 and CXCL1 release by ELISA, and ILC3 cells were extracted for qPCR analysis. **D** Western blotting analysis of GR in 16HBEs and ILC3s after stimulation with Dex (1000 ng/mL) for 15 min. **E**, **F** Western blotting analysis of phosphorylation of GR S226 and S211 in 16HBEs (**E**) and ILC3s (**F**) after stimulation with Dex (1000 ng/mL) for 15 min. **G** Ratio of p-GR S226/S211 in 16HBEs and ILC3s at baseline and after dexamethasone treatment. **H** Western blotting analysis of phosphorylation of MAPK in ILC3s with Dex stimulation (1000 ng/mL) for 15 min. **I** Effects of Dex and corresponding inhibitors on phosphorylated GR at S226 and S211 in ILC3s. **J** GR phosphorylation at S226 and S211 in ILC3s with indicated stimulation. Cells were stimulated with IL-1β (50 ng/mL) or dexamethasone (1000 ng/mL) alone or combination for 15 min. ILC3s were preincubated for 1 h with corresponding inhibitors then stimulated with dexamethasone (1000 ng/mL) for 15 min, then lysed and assessed for phosphorylation of GR S226 or GR S211 by Western blotting

Patients with neutrophilic asthma usually have poor response to corticosteroids [34–36], and our previous study has found that the numbers of ILC3s in asthma patients were not reduced after treatment with prednisolone [23]. Therefore, we speculated that ILC3s might contribute to the steroid resistance. To test this hypothesis, in vitro cultured ILC3s were treated with dexamethasone (Dex) and the expression levels of CXCL8 and CXCL1 were detected. 16HBEs were selected as reference cells, which were sensitive to dexamethasone treatment, as Dex significantly reduced CXCL8 production induced by IL-1β (Fig. 4A). We found that the CXCL8 and CXCL1 production of ILC3s in response to IL-1β was not inhibited by Dex at protein level, but partially reduced by Dex at transcriptome level (Fig. 4B, C). These findings suggest the steroid resistance of ILC3s in terms of neutrophil chemoattractants expression.

Next, we investigated the mechanisms that mediate steroids resistance in ILC3s. The effects of glucocorticoids (GCs) are mediated by their binding and activation of GC receptor (GR) [37], and phosphorylation status and sites of GR can dictate cells respond to GCs [38]. Therefore, we firstly compared the levels of GR between ILC3s and 16HBEs, and no difference was found between ILC3s and 16HBEs at baseline or after treatment of dexamethasone (Fig. 4D). Then, we measured the phosphorylation levels of two sites of GR, i.e. p-GR S226 and p-GR S211, and the results showed that the level of p-GR S226 was significantly up-regulated by Dex in both 16HBEs and ILC3s. However, there was a significant increase in the phosphorylation of GR S211 in 16HBEs and only a weak increase in ILC3s in response to Dex treatment (Fig. 4E, F and Additional file 3: Fig. S3). In addition, we also found that the ratio of p-GR S226 to p-GR S211 (p-GR S226/S211) was significantly higher in ILC3s than 16HBEs at baseline and after dexamethasone treatment (Fig. 4G). Taken together, these data indicate the steroid resistance of ILC3s is mediated by the enhanced phosphorylation at Ser226 and weak phosphorylation of GR at Ser211, with a higher ratio of p-GR S226/S211 than that of 16HBEs.

Finally, we explored the potential signaling pathways that regulate phosphorylation of GR in ILC3s. MAPK signaling pathway has been found to directly phosphorylate GR [38], and in ILC3s we found Dex treatment increased JNK MAPK phosphorylation, but not phosphorylation of p38 MAPK or Erk MAPK (Fig. 4H), which was further confirmed by the inhibitory experiment showing that the GR phosphorylation at S226 in response to dexamethasone was significantly inhibited by JNK-MAPK inhibitor SP600125 (Fig. 4I). Interestingly, we also found p65 NF-κB inhibitor (TPCA-1) also abolished the effect of Dex on phosphorylation of S226 (Fig. 4I), indicating a role of NF-κB signaling pathway in GR activation. Indeed, evidence has shown GR and NF-κB interacted with each other, dexamethasone treatment could reduce the binding of NF-kB chromatin and high levels of NF-κB attenuated GR function [39]. Since IL-1β significantly increased activity of NF-κB and JNK MAPK (Fig. 3F, G), we then sought to explore the crosstalk effects of dexamethasone and IL-1β in ILC3s. As expected, the level of GR phosphorylation at S226 was also significantly elevated by IL-1β treatment alone or combination with dexamethasone for 15 min (Fig. 4J), suggesting the crosstalk effect between dexamethasone and IL-1β in corticosteroids-insensitivity in ILC3s.

## Discussion

The pro-inflammatory roles of ILC3 in asthma AHR and inflammation have been known for several years [6], but the underlying mechanisms have not been fully illustrated. Previous studies have reported the production of IL-17 by ILC3 might be a key contributor to the airway neutrophilic inflammation through neutrophil recruitment in patients with asthma [40–42]. Here, we illustrated that percentage of ILC3s in peripheral blood of NEA patients was increased compared with EA patients and were negatively correlated with blood eosinophils. However, a phase II study with brodalumab (a human anti-IL-17 receptor monoclonal antibody) failed to show the treatment effect in subjects with moderate to severe asthma [43], which suggests other mechanisms might mediate neutrophilic inflammation in asthma. In our study, we found ILC3s produced neutrophil chemoattractants including CXCL8, CXCL1, TNF-α and GM-CSF after IL-1β stimulation, suggesting ILC3s may mediate airway neutrophilia in asthma by release of neutrophil chemoattractants. Meanwhile, we noticed a relatively high level of CXCL8 and CXCL1 released by ILC3s without stimulation, this might suggest the contribution of ILC3s in the recruitment of neutrophils in physiological condition but we could not exclude the possibility that they were due to the effect of rIL-2 in the culture medium [44, 45].

One of the clinical characteristics of patients with NEA is the poor response to corticosteroids, leading to a higher severity of disease and difficult-to-control asthma [46]. In this study, the expression of CXCL8 and CXCL1 in ILC3s upon IL-1β stimulation was insensitive to dexamethasone, suggesting that neutrophilic inflammation mediated by IL-1β-ILC3-CXCL8/CXCL1 axis may be involved in the development of steroid-resistant of asthma. To find out the mechanisms that mediate this insensitivity, we focused on the GR, which is the key receptor responsible for the physiological and pharmacological effects of glucocorticoids. GR can be phosphorylated at over 20 sites, while Ser211 and Ser226 are the two well-characterized sites. Phosphorylation at Ser211 promotes nuclear translocation and enhances transcriptional activity, while phosphorylation at Ser226 inhibits transcriptional activity and promotes nuclear export and has an inhibitory effect on GR function [38, 47]. The lack of Ser211 phosphorylation has been suggested to be a possible mechanism for GC resistance of human lymphoid cells [48]. Evidences have also shown the expression of GR Ser226 in PBMCs from severe asthma patients was significantly higher than that from non-severe asthma patients after dexamethasone treatment [49, 50]. In our study, we found the enhanced Ser226 phosphorylation while weak Ser211 phosphorylation upon dexamethasone treatment in ILC3s, and compared to GC-sensitive 16HBEs, the ratio of p-GR S226 to p-GR S211 (p-GR S226/S211) was significantly higher in ILC3s at baseline and after dexamethasone treatment, which well explained the insensitivity of ILC3s to dexamethasone treatment.

The phosphorylation of GR has been reported to be controlled by the p38 MAPK and Erk/JNK MAPK signaling pathways, of which p38 MAPK promotes the Ser211 phosphorylation while Erk/JNK MAPK down-regulates Ser211 phosphorylation and increases Ser226 phosphorylation [48, 51, 52]. In our study, we confirmed the regulatory effect of JNK MAPK on Ser226 phosphorylation by using its inhibitor SP600125, and the JNK MAPK activity was enhanced by dexamethasone treatment but not p38 MAPK activity, which reveals the intrinsic regulatory mechanisms associated with insensitivity of ILC3 to corticosteroids. Furthermore, phosphorylation of GR usually happens after binding to its ligand (GC), but a ligand-independent fashion as part of the crosstalk with other signaling pathways has also been reported [38]. A study of ligand-independent phosphorylation of GR showed TNF-α induced phosphorylation of the unliganded human GR at Ser-226 not Ser-211 [53]. In our study, we found IL-1β could induce Ser226 phosphorylation and the NF-κB inhibitor TPCA-1 demolished this effect in ILC3s. Meanwhile, IL-1β and dexamethasone could activate the same signaling pathway of JNK MAPK in ILC3s. This evidence implies that elevated IL-1β in neutrophil dominant asthma may have a crosstalk effect on dexamethasone [54], which aggravates corticosteroids insensitivity in neutrophilic asthma [55]. However, we could not exclude the possibility that the IL-1β induced phosphorylation at Ser226 might be through TNF-α, which has been shown to impair glucocorticoid receptor phosphorylation at Ser211 [56, 57] and directly phosphorylate GR at Ser226 by activating JNK MAPK [58], because in our study, TNF-α was enhanced by IL-1β in ILC3s.

However, there are several limitations that need to be addressed in this study. Firstly, due to the COVID-19 pandemic, we were unable to obtain induced sputum samples from asthma patients, thus unable to detect ILC3 in airways and distinguish neutrophilic asthma from NEA; therefore, EA and NEA were classified according to blood eosinophil counts 300/μL. Secondly, different subgroups of ILC3 have not been investigated, which could provide more information of ILC3 heterogeneity. Third, we did not directly compare the stimulation responses of ILC3 from healthy control and different asthma phenotypes/severities, which might imply the functional differences of ILC3 in the context of disease and asthma phenotypes. Fourth, we did not investigate other potential mechanisms that could be involved in the regulation of glucocorticosteroids such as GR isoforms (GRβ) and the lack of GRE in CXCL8 gene. Finally, we focused on patients with asthma, however, it would be interesting to investigate the roles of ILC3 in a broader spectrum of diseases such as patients with chronic obstructive pulmonary disease (COPD) as they are generally less sensitive to inhaled steroids and age dependent, and evidence showed ILC3s were notably increased in lung tissue from patients with COPD [59].

In conclusion, ILC3s are elevated in patients with NEA compared to EA and are crucial contributors to the neutrophilic airway inflammation via production of neutrophil chemoattractants through p65 NF-κB and p38/JNK MAPK signaling pathways. Specifically, the expression of neutrophil chemoattractants induced by IL-1β is insensitive to dexamethasone, which is due to aberrant GR phosphorylation with increased phosphorylation at Ser226 and weak phosphorylation at Ser211. Our findings provide evidence of the roles of innate immunity in neutrophilic inflammation and steroids insensitivity, which help to discover novel treatment targets for future drug development and better management of neutrophilic phenotype of asthma.

## Supplementary Information

**Additional file 1: Figure S1.** Flow cytometric gating strategy for ILCs in PBMCs. ILC2 populations were gated as Lin^−^CD45^+^CD3^−^CD4^−^CD8^−^ CD127^+^CRTH2^+^, ILC1 populations were gated as Lin^−^CD45^+^CD3^−^CD4^−^CD8^−^ CD127^+^CRTH2^−^CD117^−^, and ILC3 populations were gated as Lin^−^CD45^+^CD3^−^CD4^−^CD8^−^CRTH2^−^CD127^+^CD117^+^ Lineage markers contained CD11b, CD11c, CD14, CD19, CD56, CD123, and FcεRI. ILCs, innate lymphoid cells; ILC1, group 1 innate lymphoid cell; ILC2, group 2 innate lymphoid cell; ILC3, group 3 innate lymphoid cell.

**Additional file 2: Figure S2.** A, Blood neutrophils count and percentage among healthy control (HC) and patients with NEA and EA. B, Correlation between ILC subsets and blood neutrophils count and percentage. **** p < 0.0001, *** p < 0.001, ** p < 0.01, * p < 0.05, ns p > 0.05.

**Additional file 3: Figure S3.** A, Level of p-GR S226 and p-GR S211 in HBEs with or without dexamethasone treatment. B, Level of p-GR S226 and p-GR S211 in ILC3s with or without dexamethasone treatment.

**Additional file 4: Table S1.** Antibodies used in flow cytometry and cell sorting. **Table S2.** Primer sequences used in RT-PCR. **Table S3.** Antibodies used for western blotting.

## Data Availability

The datasets used and/or analysed during the current study are available from the corresponding author on reasonable request.

## Abbreviations

ACT: Asthma Control Test
AHR: Airway hyperreactivity
BAL: Bronchoalveolar lavage
BCA: Bicinchoninic acid
BMI: Body mass index
BP: Biological process
CC: Cellular component
CXCL: C-X-C motif ligand
Dex: Dexamethasone
DNA: Deoxyribonucleic acid
EA: Eosinophilic asthma
ELISA: Enzyme-linked immunosorbent assay
Erk: Extracellular-signal-regulated kinase
FeNO: Fractional exhaled nitric oxide
GC: Glucocorticoid
GINA: Global Initiative for Asthma
GM-CSF: Granulocyte–macrophage colony-stimulating factor
GO: Gene Ontology
GR: Glucocorticoid receptor
16HBE: Human bronchial epithelial cell line
HC: Healthy control
IFN: Interferon
IgE: Immunoglobulin E
IL: Interleukin
ILC: Innate lymphoid cell
ILC1: Group 1 innate lymphoid cell
ILC2: Group 2 innate lymphoid cell
ILC3: Group 3 innate lymphoid cell
IRB: Institutional Review Board
JNK: C-jun N-terminal kinase
MAPK: Mitogen-activated protein kinase
MF: Molecular function
MMP-9: Matrix metalloprotease 9
mRNA: Messenger Ribose Nucleic Acid
NE: Neutrophil elastase
NEA: Non-eosinophilic asthma
NF-kB: Nuclear factor kB
PBMC: Peripheral blood mononuclear cell
PCR: Polymerase chain reaction
PMA: Phorbol 12-myristate 13-acetate
PMSF: Phenylmethanesulfonyl fluoride
RIPA: Radio immunoprecipitation
RNA: Ribose Nucleic Acid
RORγt: Retinoid-related orphan receptorγt
RT-PCR: Real-time polymerase chain reaction
SD: Standard deviation
Th17 cells: T helper 17 cells
TNF: Tumor necrosis factor
