# Supplementary figures and images for: Group 3 innate lymphoid cells secret neutrophil chemoattractants and are insensitive to glucocorticoid via aberrant GR phosphorylation
Source: Respir Res. 2023 Mar 23;24:90. doi: 10.1186/s12931-023-02395-5 (PMC10033286; doi:10.1186/s12931-023-02395-5)

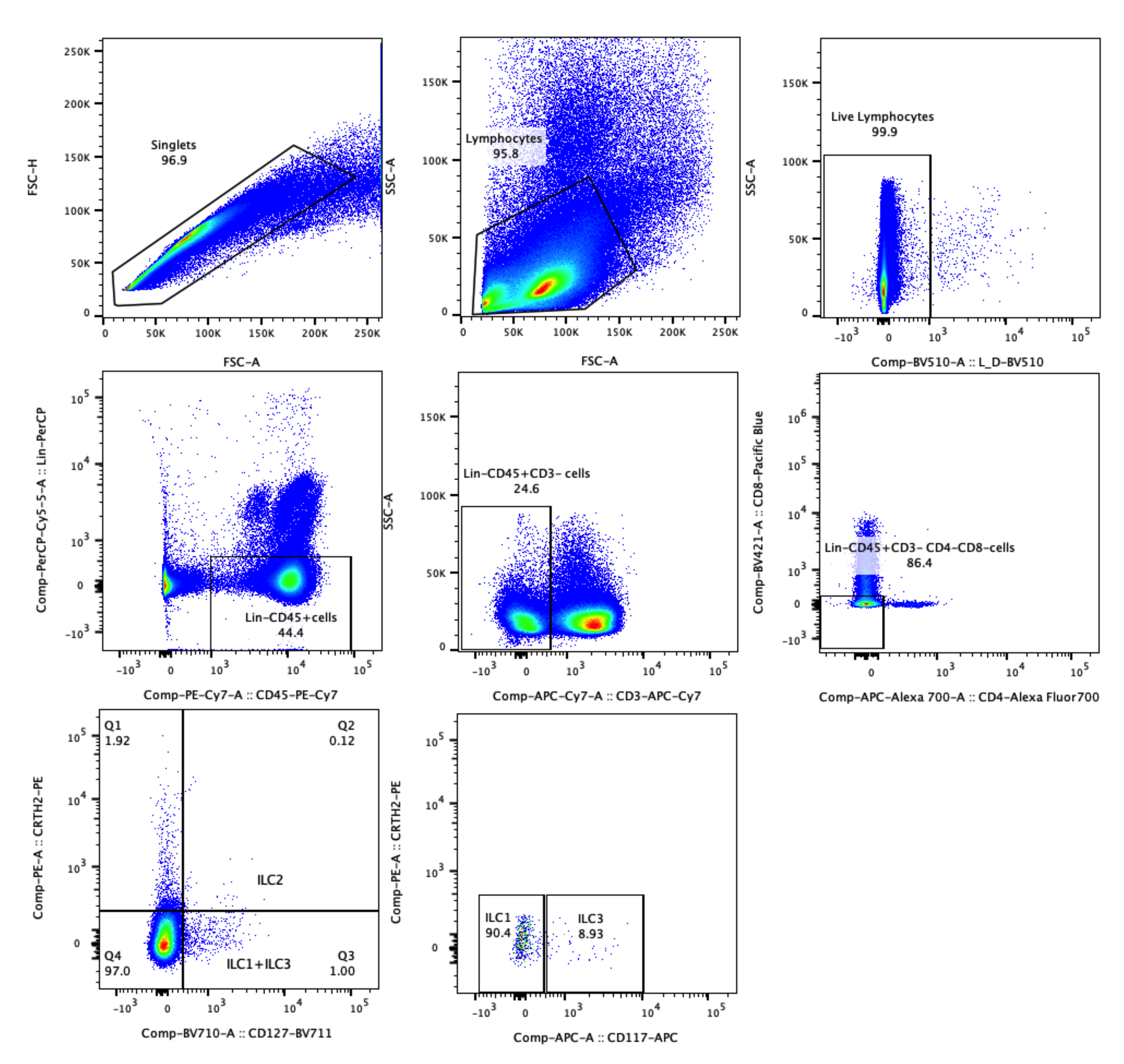

Supplement: Supplementary file 1 — Additional file 1: Figure S1. Flow cytometric gating strategy for ILCs in PBMCs. ILC2 populations were gated as Lin−CD45+CD3−CD4−CD8− CD127+CRTH2+, ILC1 populations were gated as Lin−CD45+CD3−CD4−CD8− CD127+CRTH2−CD117−, and ILC3 populations were gated as Lin−CD45+CD3−CD4−CD8−CRTH2−CD127+CD117+ Lineage markers contained CD11b, CD11c, CD14, CD19, CD56, CD123, and FcεRI. ILCs, innate lymphoid cells; ILC1, group 1 innate lymphoid cell; ILC2, group 2 innate lymphoid cell; ILC3, group 3 innate lymphoid cell. [file 12931_2023_2395_MOESM1_ESM.tif]

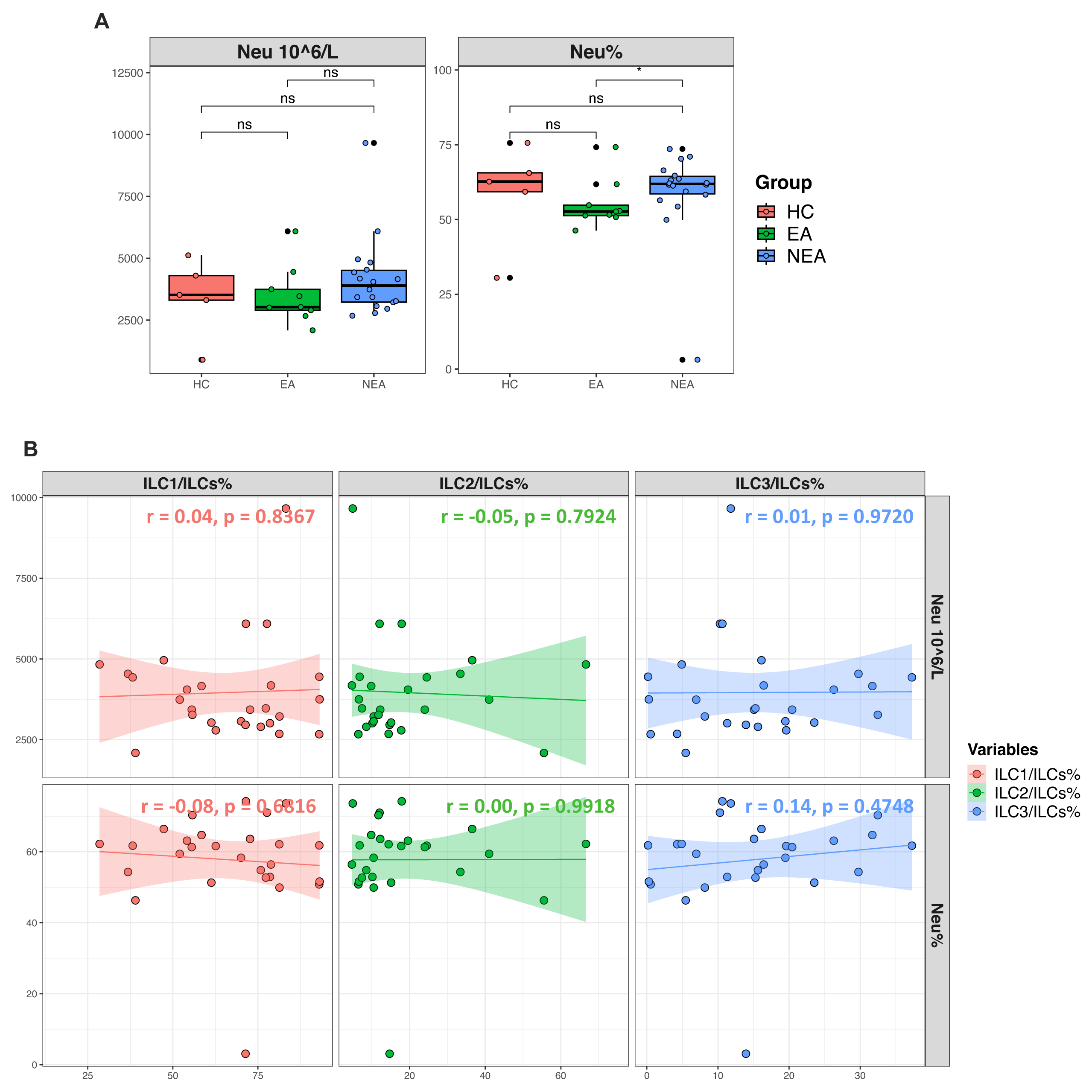

Supplement: Supplementary file 2 — Additional file 2: Figure S2. A, Blood neutrophils count and percentage among healthy control (HC) and patients with NEA and EA. B, Correlation between ILC subsets and blood neutrophils count and percentage. **** p < 0.0001, *** p < 0.001, ** p < 0.01, * p < 0.05, ns p > 0.05. [file 12931_2023_2395_MOESM2_ESM.tif]

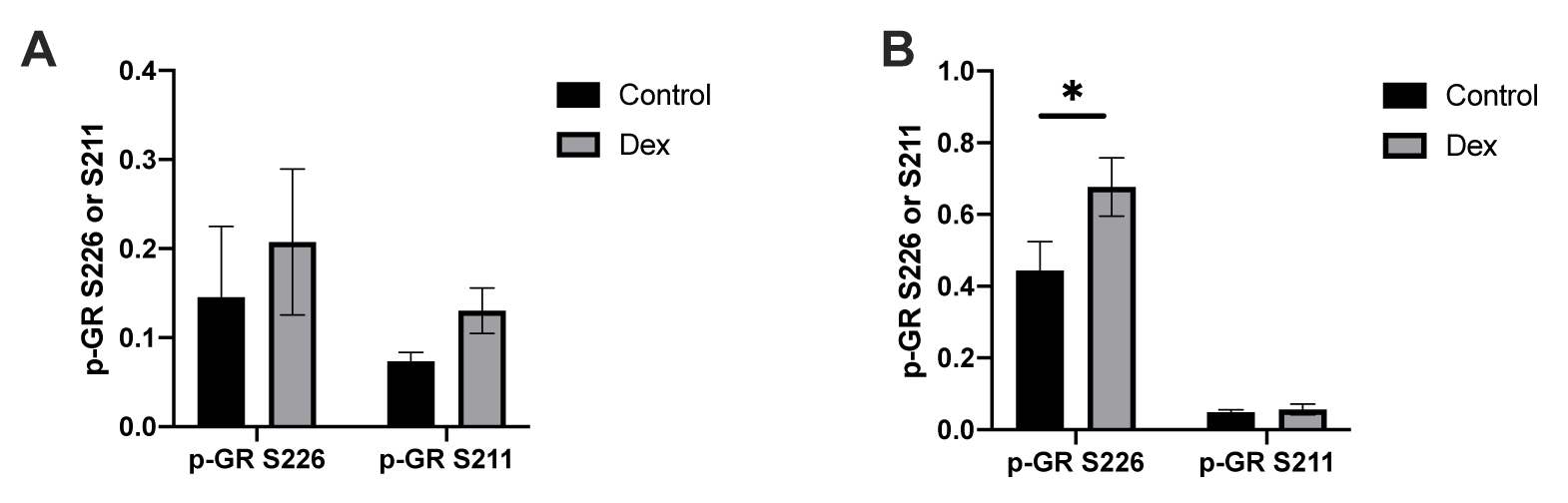

Supplement: Supplementary file 3 — Additional file 3: Figure S3. A, Level of p-GR S226 and p-GR S211 in HBEs with or without dexamethasone treatment. B, Level of p-GR S226 and p-GR S211 in ILC3s with or without dexamethasone treatment. [file 12931_2023_2395_MOESM3_ESM.tif]
