# Supplementary material for: Group 3 innate lymphoid cells secret neutrophil chemoattractants and are insensitive to glucocorticoid via aberrant GR phosphorylation
Source: Respir Res. 2023 Mar 23;24:90. doi: 10.1186/s12931-023-02395-5 (PMC10033286; doi:10.1186/s12931-023-02395-5)
Supplement: Supplementary file 4 — Additional file 4: Table S1. Antibodies used in flow cytometry and cell sorting. Table S2. Primer sequences used in RT-PCR. Table S3. Antibodies used for western blotting. [file 12931_2023_2395_MOESM4_ESM.docx]

**Group 3 innate lymphoid cells secret neutrophil chemoattractants and are insensitive to glucocorticoid via aberrant GR phosphorylation**

Li Xiu He^*^, Ling Yang^*^, Ting Liu, Yi Na Li, Ting Xuan Huang, Lan Lan Zhang, Jian Luo^#^, Chun Tao Liu^#^.

**S-Tables**

| Table S1 Antibodies used in flow cytometry and cell sorting | | | |
| --- | --- | --- | --- |
| Antigen | Clone | Supplier | Use |
| CD3 | OKT3 | BioLegend | Flow cytometry (Panel 1/2/3) |
| CD4 | OKT4 | BioLegend | Flow cytometry (Panel 1/2) |
| CD8 | SK1 | BioLegend | Flow cytometry (Panel 1/2) |
| CD11b | DCIS1/18 | Abcam | Flow cytometry (Panel 1/2) |
| CD11c | BU15 | BioLegend | Flow cytometry (Panel 1/2) |
| CD14 | MφP9 | BD Biosciences | Flow cytometry (Panel 1/2) |
| CD19 | 4G7 | BD Biosciences | Flow cytometry (Panel 1/2) |
| CD45 | H130 | BioLegend | Flow cytometry (Panel 1/2) |
| CD56 | HCD56 | BioLegend | Flow cytometry (Panel 1/2) |
| CD117 | 104D2 | eBioscience | Flow cytometry (Panel 1/2) |
| CD123 | 32703 | R&D Systems | Flow cytometry(Panel 1/2) |
| CD127 | A019D5 | BioLegend | Flow cytometry (Panel 1/2) |
| CRTH2 | BM16 | Miltenyi | Flow cytometry (Panel 1/2) |
| FcεRI | AER-37 | BioLegend | Flow cytometry (Panel 1/2) |
| RORγt | AFKJS-9 | Bioscience | Flow cytometry |
| IL-8/CXCL8 | BH0814 | Bioscience | Flow cytometry |
| IL-17A | N49-653 | BD Biosciences | Flow cytometry |
| IL-22 | 2G12A41 | BioLegend | Flow cytometry |

| Table S2 Primer sequences used in RT-PCR | | |
| --- | --- | --- |
| Gene name (abbreviation) |  | Primer sequence |
| CXCL8 | Forward  Reverse | 5′-AACTGAGAGTGATTGAGAGTGG-3′  5′-ATGAATTCTCAGCCCTCTTCAA-3′ |
| CXCL1 | Forward  Reverse | 5′-AAGAACATCCAAAGTGTGAACG-3′  5′-CACTGTTCAGCATCTTTTCGAT-3′ |
| TNF-α | Forward  Reverse | 5′-GTGACAAGCCTGTAGCCCAT-3′  5′-CTCTGATGGCACCACCAACT-3′ |
| GM-CSF | Forward  Reverse | 5′-GGTCATCTTGGAGGGACCAA-3′  5′-TGCCATGCCTGTATCAGGGT-3′ |

| Table S3 Antibodies used for western blotting | | | | |
| --- | --- | --- | --- | --- |
| Antibody | Source | Dilution | Lot number | Supplier |
| Anti-NF-κB p65 | rabbit IgG | 1:1000 | D14E12 | CST |
| Anti-Phospho-NF-κB p65 | rabbit IgG | 1:1000 | 93H1 | CST |
| Anti-p38 | rabbit IgG | 1:1000 | ab170099 | Abcam |
| Anti-Phospho-p38 MAPK | rabbit IgG | 1:1000 | D3F9 | CST |
| Anti-Erk1/2 | rabbit IgG | 1:10000 | ab184699 | Abcam |
| Anti-p-Erk1/2 | rabbit IgG | 1:1000 | ab201015 | Abcam |
| Anti-JNK | rabbit IgG | 1:1000 | ab179461 | Abcam |
| Anti-p-JNK | rabbit IgG | 1:1000 | ab124956 | Abcam |
| Anti-GR | rabbit IgG | 1:2000 | ab183127 | Abcam |
| Anti-GR (phospho S226) | rabbit IgG | 1:1000 | ab228972 | Abcam |
| Anti-GR (phospho S211) | rabbit IgG | 1:1000 | AP0759 | ABclonal, China |
| Anti-GAPDH | rabbit IgG | 1:10000 | ab181602 | Abcam |
| NF-κB, Nuclear factor kB; MAPK, Mitogen-activated protein kinase; Erk, Extracellular-signal-regulated kinase; JNK, c-jun N-terminal kinase; GR, Glucocorticoid receptor; GAPDH, Glyceraldehyde-3-phosphate dehydrogenase; CST, Cell Signaling Technology. | | | | |

**S-FIGURE LEGENDS**

**Figure S1.** Flow cytometric gating strategy for ILCs in PBMCs. ILC2 populations were gated as Lin^-^CD45^+^CD3^-^CD4^-^CD8^-^ CD127^+^CRTH2^+^, ILC1 populations were gated as Lin^-^CD45^+^CD3^-^CD4^-^CD8^-^ CD127^+^CRTH2^-^CD117^-^, and ILC3 populations were gated as Lin^-^CD45^+^CD3^-^CD4^-^CD8^-^CRTH2^-^CD127^+^CD117^+^ Lineage markers contained CD11b, CD11c, CD14, CD19, CD123, and FcεRI. ILCs, innate lymphoid cells; ILC1, group 1 innate lymphoid cell; ILC2, group 2 innate lymphoid cell; ILC3, group 3 innate lymphoid cell.

**Figure S2.** A, Blood neutrophils count and percentage among healthy control (HC) and patients with NEA and EA. B, Correlation between ILC subsets and blood neutrophils count and percentage. **** p<0.0001, *** p<0.001, ** p<0.01, * p<0.05, ns p>0.05.

**Figure S3.** A, Level of p-GR S226 and p-GR S211 in HBEs with or without dexamethasone treatment. B, Level of p-GR S226 and p-GR S211 in ILC3s with or without dexamethasone treatment.
